# Impact of adolescent internet addiction on academic procrastination: the serial mediating role of self-control and anxiety

**DOI:** 10.3389/fpsyg.2025.1713213

**Published:** 2025-12-19

**Authors:** Zhaoliang Wu, Li Xue, Yingxue Zhang, Fengjin Zhan, Hanmo Li, Ruici Liu, Xi Yang, Zi Chen

**Affiliations:** 1 Chengdu Medical College, Chengdu, Sichuan, China; 2 Chengdu Zongnan Hospital, Chengdu, Sichuan, China; 3 Ruoshui Middle School, Liangshan, Sichuan, China

**Keywords:** academic procrastination, anxiety, college student, high school students, internet addiction, self control

## Abstract

**Introduction:**

This study investigated the mediating roles of self-control and anxiety in the relationship between Internet addiction and academic procrastination. Aiming to understand the psychological state of academic procrastinators and provide theoretical reference for taking effective intervention measures.

**Methods:**

Data were collected from a sample of 2,421 adolescents from both a high school and university in Southwest China by convenience sampling. Measures used included the Demographic Information Questionnaire, Internet Addiction Test, Aitken Procrastination Inventory, Self-Control Scale, and Self-Rating Anxiety Scale.

**Results:**

Internet addiction significantly negatively predicted self-control (*β* = −0.552, *p* < 0.001) and significantly positively predicted anxiety (*β* = 0.244, *p* < 0.001) and academic procrastination (*β* = 0.214, *p* < 0.001). Self-control significantly negatively predicted both anxiety (*β* = −0.249, *p* < 0.001) and academic procrastination (*β* = −0.430, *p* < 0.001). Anxiety significantly positively predicted academic procrastination (*β* = 0.082, *p* < 0.001).

**Conclusion:**

The study found that Internet addiction not only has a direct effect on academic procrastination, but also self-control and anxiety can partially mediate the relationship between Internet addiction and academic procrastination. In addition, self-control and anxiety also play a serial mediating role in the relationship between internet addiction on academic procrastination.

## Introduction

Learning is the primary way to acquire knowledge and information. With the development of society, the speed of knowledge updating and replacement is accelerating, and learning has become particularly important. Although there are more methods of learning with the advancement of technology, the learning problems presented at the same time are also more diverse, among which academic procrastination has been proven to be a common phenomenon among teenagers.

Academic procrastination (AP) refers to the behavioral tendency to avoid or delay academic tasks within designated timeframes, often initiating work only as deadlines approach (Ji, 2016). This phenomenon is prevalent among adolescents worldwide, with studies indicating that 70–90% of students procrastinate over assignments (Soleimani et al., 2023). Pang and Han (2009) found that 69–90% of Chinese university students experienced negative consequences because of AP. This behavior can seriously affect the academic progress and mental health of adolescents.

Multiple factors contribute to AP. In the current era of widespread mobile device use, the convenience and entertainment provided by the Internet have exacerbated AP among adolescents. The relationship between Internet addiction (IA) and AP has garnered increasing research attention. IA is defined as excessive or pathological use of Internet-based technologies (Yan, 2021). IA is a prevalent behavioral problem among adolescents, with reported prevalence rates ranging from 9 to 38% (Malakeh, et al., 2017). Excessive Internet use inevitably displaces time devoted to academic activities, thereby leading to procrastination. Therefore, this study proposes hypothesis H1: IA has a direct predictive effect on AP.

IA can lead to a series of behavioral dyscontrol and emotional reactions (Pu and Ding, 2025). Previous studies have demonstrated that a direct consequence of IA is a decline in self-control, which in turn results in negative emotions such as anxiety due to an inability to regulate internet usage time (Shi et al., 2020). Bandura’s (1991) self-regulation theory provides an explanation. This theory posits that individuals regulate their psychological and behavioral states through three processes: self-monitoring, self-evaluation, and self-reaction. Specifically, individuals monitor their psychological and behavioral states, and assess whether these states meet their expectations, and thus experience various psychological phenomena, including emotional reactions. According to this theory, the relationship among these three factors can be explained as follows: because individuals fail to effectively control their internet usage time (i.e., self-regulation failure), they perceive their psychological and behavioral states as deviating from individuals’ expectations, which leading to intense negative emotions such as anxiety. Additionally, Christopher’s (2003) study explored the relationship between academic procrastination and self-regulation from the perspective of self-regulation theory, concluding that academic procrastination is a result of self-regulatory failure.

Self-control (SC), which is defined as an individual’s ability to manage impulses, emotions, and behaviors to achieve long-term goals (Yang et al., 2012). Regarding the relationship between IA, SC and AP Zhang et al. (2024) study found that IA excessively depletes adolescents’ resources, including SC, that are essential for academic goal attainment. The depletion of SC resources increases academic pressure, making task completion difficult. Ultimately, this behavior results in procrastination. Kim et al.’ (2017) study about Korean university students found that lower SC predicted greater procrastination severity. Similarly, Aknci’s (2021) study of Turkish students demonstrated a significant negative correlation between SC and AP. Conversely, Saunders et al. (2018) demonstrated that individuals with strong SC are more effective at overcoming academic challenges. Therefore, this study proposes hypothesis H2: SC partially mediate the relationship between IA and AP.

A large amount of preliminary studies have confirmed that IA significantly and positively predicts anxiety(AN) and that excessive Internet use reduces offline social interactions, thereby exacerbating stress and anxiety (Stanković and Nešić, 2022). Steel and Klingsieck (2016) identified AN as core incentives to all types of procrastination. This study indicates that AN can exacerbate AP. As Sirois and Pychyl’s (2013) study of short-term mood repair mechanism considers that individuals prioritize immediate emotional relief (e.g., anxiety reduction) over long-term goals, ultimately hindering academic processes. Sun’s (2023) study explains the mental activity of academic procrastinators: individuals first evaluate the task; if they find it difficult to complete a task, they may experience negative emotions such as anxiety. Therefore, to avoid negative emotions, individuals choose to do something easier, leading to procrastination. Other studies have also suggested that individuals with anxiety are more likely to have AP; for instance, test-anxious students experience negative somatic symptoms and avoidance tendencies that promote procrastination (Custer, 2018; Krispenz et al., 2019). Therefore, this study proposes hypothesis H3: AN partially mediate the relationship between IA and AP.

Gao (2021) has conducted a prospective study on the influence and mechanism of IA, SC and AN on AP. The study show that Internet addiction will impair their SC, and then make individuals experience negative emotions such as anxiety. This study reflects the reduction of SC, which will further make individuals experience AN. Combined with the above, the reduction of self-control ability aggravates the anxiety of individuals, which ultimately increases the possibility of academic procrastination. Therefore, this study proposed hypothesis H4: SC and AN play a serial mediating role in the relationship between IA and AP.

**Figure 1:**
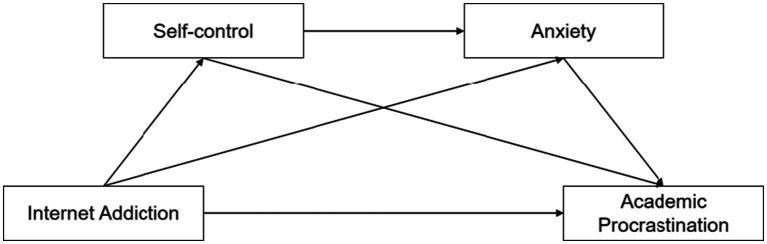
Theoretical model of internet addiction, self-control, anxiety, and academic procrastination.

In summary, previous research has indicated that IA, SC, and AN significantly influence AP. In addition, these variables have interconnected mechanisms. However, the specific role and mechanism of the most common behavioral and emotional responses in the mechanism of academic procrastination caused by internet addiction have not been fully validated. Therefore, this study proposed a theoretical model (Figure 1) to examine the mediating roles of SC and anxiety in the relationship between adolescent IA and AP and explored the underlying mechanisms of AP, aiming to understand the psychological state of academic procrastinators and provide theoretical reference for taking effective intervention measures. Based on the literature review above, the following hypotheses are proposed:

*H1*: IA has a direct effect on AP.

*H2*: SC mediates the relationship between IA and AP.

*H3*: AN mediates the relationship between IA and AP.

*H4*: IA play a serial mediating role in the relationship between IA and AN.

## Methods

### Design and participants

**Figure 2:**
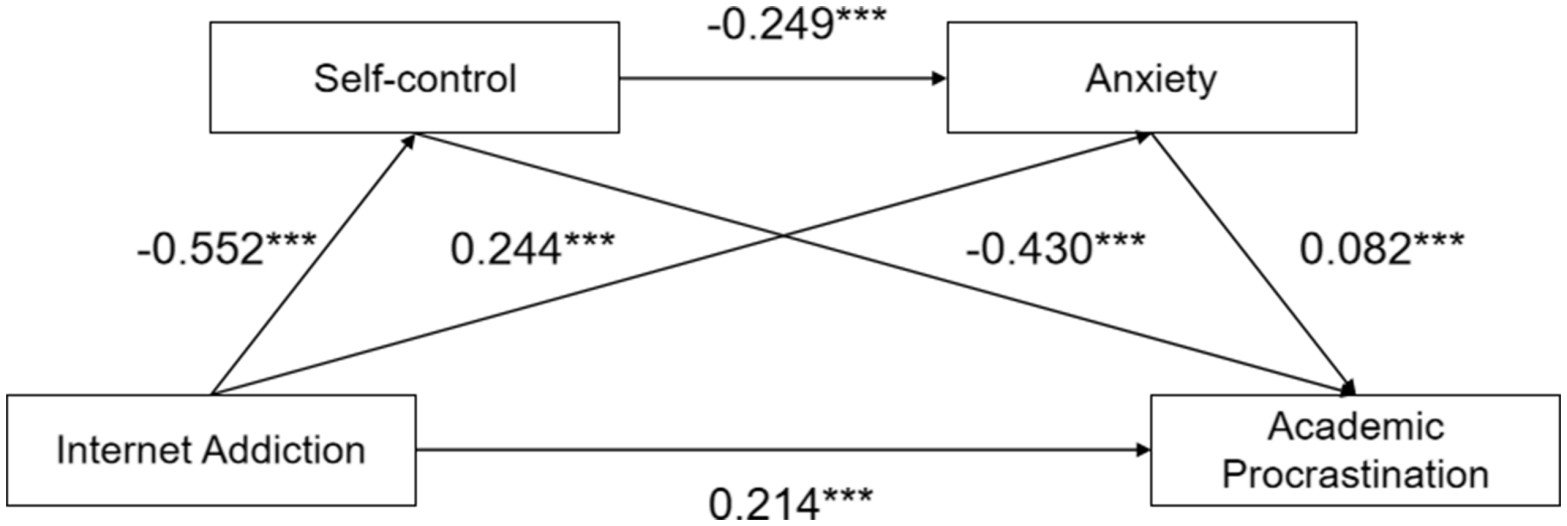
Theoretical model of internet addiction, self-control, anxiety, and academic procrastination. ^***^p < 0.01.

The study has a cross-sectional explanatory design and collecting data by convenience sampling. The study establish the relationships between the variables via statistical correlation tests. The participants were adolescents from both a high school and university in Southwest China. A total of 2,650 adolescents participated in this study. In addition, 2,421 valid responses were retained after excluding invalid submissions, resulting in a valid response rate of 91.35%. The sample comprised 1,029 males (*M* = 16.89 years) and 1,389 females (*M* = 17.05 years), with no significant age difference between genders (*p* > 0.05). The participants’ age ranged from 14 to 24 years (*M* = 16.98 years) (Figure 2).

### Procedure

The study was approved by the ethics committee of Chengdu Medical College Biomedical Ethics Committee. All participants have be obtained informed consent forms after they were informed of the survey’s purpose and emphasize its confidentiality. Researchers distributed and collected questionnaires on the spot during break between classes. The questionnaires were completed anonymously throughout the process and collected uniformly after filling.

### Measures

#### Demographic information questionnaire

The instruments included a demographic information questionnaire survey. The demographic information included variables such as gender, age, and grade level.

The psychological measures used in this study include the following.

#### Internet Addiction Test

This study used the Internet Addiction Test developed by Young (2017) to assess adolescent IA. This 20-item instrument employs a 5-point Likert scale (1 = strongly disagree, 5 = strongly agree). The total scores were calculated by summing all items, with higher scores indicating a greater tendency toward IA. In the Chinese version study by Lu et al. (2019), the Cronbach’s *α* = 0.927. After factor analysis, the correlation coefficients between each item and the total score were above 0.5, and the factor loadings of all items exceeded 0.4, indicating that the scale has good psychometric properties. In this study (Cronbach’s *α* = 0.891), the scale demonstrated good internal consistency.

#### Aitken Procrastination Inventory

API developed by Aitken in 1982. This study used the Chinese version revised by Chen et al. (2008). This unidimensional self-report scale consists of 19 items rated on a 5-point Likert scale (1 = completely disagree, 5 = completely agree). Higher total scores indicated more severe procrastination behaviors. The Cronbach’s *α* = 0.802, and the criterion validity is calculated using the product difference correlation method to determine the correlation between the total score of this scale and the average number of days completed, with a correlation coefficient of 0.727. Therefore, it is considered to have good psychometric properties. In this study (Cronbach’s *α* = 0.716), the scale demonstrated acceptable internal consistency.

#### Self-Control Scale

SCS developed by Tanney in 2004. This study used the Chinese version of the Self-Control Scale revised by Tan and Guo (2008). This unidimensional scale consists of 19 items rated on a 5-point Likert scale (1 = completely disagree, 5 = completely agree), with items 1, 5, 11, and 14 reverse-scored. Higher total scores indicated greater SC. The Cronbach’s *α* = 0.862, and the total variability explained by the five dimensions reaches 53.7%, indicating good psychometric properties. In this study (Cronbach’s α = 0.865), the scale demonstrated good internal consistency.

#### Self-Rating Anxiety Scale

The Self-Rating Anxiety Scale, developed by Zung, assesses individuals’ subjective anxiety experiences by focusing on symptom frequency (Wang, 1999). This unidimensional, 20-item instrument uses a 4-point rating scale (1 = none or a little of the time, 2 = some of the time, 3 = a good part of the time, and 4 = most or all of the time). Standard scores indicated anxiety intensity, with higher scores indicating greater symptom severity. In Tao and Gao (1994) study, the Cronbach’ α = 0.931, and after structural validity analysis, it was found that there were five factors that could well reflect the validity of the scale. In this study (Cronbach’s α = 0.820), the scale demonstrated acceptable internal consistency.

### Statistical analysis

Statistical analyses were performed using IBM SPSS version 22.0 for data management and processing. The analytical methods included (1) descriptive statistics, including the mean, standard deviation, and normality tests. (2) Pearson correlation analysis, (3) path analysis with a serial mediation model (Hayes, 2013), and (4) the bias-corrected percentile bootstrap method with 5,000 resamples to decompose the mediation effects. Statistical significance was set at *p* < 0.05 (two-tailed). In addition, the model was primarily evaluated based on the values of the fit indices: when GIF, and CFI > 0.95, RMSEA < 0.05 the model is considered a good model (Wen and Hou, 2004). Internet Addiction is the predictor variable; Both Self-control and Anxiety are mediator variables; Academic procrastination is the result.

## Results

### Assessment of common method variance

Given that all data were collected via self-reported questionnaires, we first assessed common method variance using Harman’s single-factor test. The analysis yielded 16 factors with eigenvalues greater than 1.0, with the first factor accounting for 17.71% of the total variance—below the 40% threshold—indicating no significant common method bias in this study (Zhou and Long, 2004).

### Correlation analysis of variables

The measures of skewness and kurtosis in Table 1 were found in a normal distribution range (±1.5). Following Kline’s study for large samples (|skewness| ≤ 3, |kurtosis| ≤ 10), these values indicate an approximately normal distribution (Bai et al., 2025).

**Table 1:** Descriptive statistics and correlation analysis of the overall sample (*N* = 2,421).

Variables	M	SD	S	K	1	2	3
1 IA	47.50	12.79	0.91	1.16	–		
2 AP	49.68	8.44	0.48	0.28	0.48^**^	–	
3 SC	57.36	11.12	0.13	0.45	−0.58^**^	−0.38^**^	–
4 AN	43.83	9.84	−0.09	−0.13	0.33^**^	0.38^**^	−0.55^**^

^***^p < 0.001, ^**^p < 0.01, ^*^p < 0.05.

IA, internet addiction; AP, academic procrastination; SC, self-control; AN, anxiety.

Pearson product–moment correlation analysis was performed for all the independent, mediator, and dependent variables. The results of the correlation analysis between perceived AP, anxiety, IA, and SC are presented in Table 1.

The results of the Pearson product–moment correlation analysis presented in Table 1 indicate that AP was significantly and positively correlated with IA (*r* = 0.48, *p* < 0.01) and AN (*r* = 0.38, *p* < 0.01); IA was significantly and positively correlated with AN (*r* = 0.33, *p* < 0.01); and SC was negatively correlated with AP (*r* = −0.38, *p* < 0.01), IA (*r* = −0.58, *p* < 0.01), and AN (*r* = −0.055, *p* < 0.01).

### Examination of the serial mediating roles of SC and AN in the relationship between IA and AP

To avoid saturation of the model, this study used a structural residual model to test the model fit: GIF = 0.992, CFI = 0.987, and RMSEA = 0.037. Therefore, it can be considered a good model.

Following Zhao et al.’s (2024) study, all variables were standardized to mitigate multicollinearity. The analysis was performed using Model 6 of the SPSS PROCESS macro (a serial mediation model) with bias-corrected percentile bootstrap estimation. As shown in Table 2, analysis of standardized path coefficients showed that IA negatively affected SC (*β* = −0.552, *p* < 0.001) and positively affected AN (*β* = 0.244, *p* < 0.001) and AP (*β* = 0.214, *p* < 0.001). SC negatively affected AN (*β* = −0.249, *p* < 0.001) and AP (*β* = −0.430, *p* < 0.001), while AN positively affected AP (*β* = 0.082, *p* < 0.001).

**Table 2:** Regression analysis of the mediating role (*N* = 2,421).

Variables	SC		AN		AP	
	*β*	*t*	*β*	*t*	*β*	*t*
IA	−0.552	−33.334^***^	0.244	11.287^***^	0.214	11.479^***^
SC			−0.249	−11.490^***^	−0.430	−22.499^***^
AN					0.082	7.830^**^
R^2^	0.306		0.193		0.395	
*F*	533.60^***^		192.672^***^		394.771^***^	

^**^p < 0.01, ^***^p < 0.001.

Further mediation analyses using the bias-corrected percentile bootstrap method (5,000 resamples) revealed the following results. IA had a positive total direct effect on AP (effect = 0.224, 95% CI [0.186, 0.262]), accounting for 45.43%. IA had a total indirect effect on AP through SC (effect = 0.241, 95% CI [0.213, 0.270]) and AN (effect = 0.018, 95% CI [0.009, 0.028]) and a serial indirect effect through SC and AN (effect = 0.010, 95%, CI [0.005, 0.015]), accounting for 54.56%. Notably, all the bootstrap 95% confidence intervals included 0 (zero), confirming the statistical significance of the mediation effects across each pathway (Table 3).

**Table 3:** Decomposition of intermediary effects (*N* = 2,421).

Effect	Relationship	Estimate	VAF	CI (95%)
Direct effect	IA → AP	0.224	45.43%	[0.186, 0.262]
Indirect effect	IA → SC → AP	0.241	42.59%	[0.213, 0.270]
	IA → AN→AP	0.018	3.65%	[0.009, 0.028]
	IA → SC → AN→AP	0.010	2.03%	[0.005, 0.015]
Total indirect effect		0.269	54.56%	[0.240, 0.299]
Total effect		0.493	100%	[0.458, 0.527]

## Discussion

The findings of this study demonstrated that IA significantly and positively predicted AP, consistent with the findings of previous studies (Edwin et al., 2023; Bian, 2023). This indicates that Internet addictive behaviors constitute a disruptive factor that contributes to students’ AP (Karakaya and Altinsoy, 2023). In other words, the more severe the tendency toward IA among students, the more severe their AP. Students may persistently engage in online behavior, making them fall into various temptations, and subsequently manifest AP. It is necessary for families and schools to closely monitor the internet usage of teenagers, take effective intervention in their internet usage behavior as early as possible, and avoid serious impact on their studies.

Consistent with our hypotheses, SC partially mediated the relationship between IA and AP. IA had both direct and indirect effects on procrastination through diminished SC. The result is consistent with previous study results and the hypothesis of this study (Ma, 2024; Yang et al., 2019). Li’s (Li and Tian, 2025) research also shows that IA can lead to decreased SC and inevitably affects individuals’ normal life, making it difficult to control impulses, resist temptations, and achieve goals and ultimately resulting in AP. This result proves that self-control is an important variable in shaping adolescent health behavior. It is necessary for schools to carry out self-control training to enhance students’ self-control (Li et al., 2025).

Consistent with the hypothesis of this study, anxiety partially mediated the impact of IA on AP. IA can cause individuals to experience anxiety, leading to AP. Consistent with Bian’s (2023) results, mobile phone dependence also belongs to a type of internet addiction, and the occurrence of academic procrastination can be partially attributed to negative emotions such as anxiety. Moreover, this study validated the short-term emotional repair mechanism (Sirois and Pychyl, 2013). If the rewards related to the task are long term or the task has unpleasant characteristics, such as boredom or difficulty, individuals may experience negative emotions related to the task, such as anxiety. Focusing on regulating emotions in the short term cannot suppress the impulse to avoid a task and may lead to procrastination (Custer, 2018). This suggests that adolescents with IA are likely to exhibit anxiety, which is also one of the psychological mechanisms behind AP and requires close attention from home and school.

The most valuable finding of this study is that IA had a serial indirect effect on AP through SC and anxiety. This result is consistent with Gao’s (2021) study, which suggests that internet addiction can lead to decreased self-control, which in turn can result in anxiety. This also validates the self-regulation theory (Bandura, 1991), which states that IA leads individuals to lose control of their behavior. Subsequently experience strong anxiety when evaluating that their behavior did not meet expectations. Huang believes that IA shifts individuals’ attention from learning to the online world, and in this process, addictive behavior damages brain regions related to self-regulation, affecting individuals’ SC (Huang, 2023). Therefore, they cannot effectively regulate their anxiety. Moreover, it can lead to negative emotions. Therefore, individuals alleviate their anxiety by avoiding academic tasks, leading to AP. This result validates Christopher’s (2003) study, which proves that individuals who fail to regulate their own psychology and behavior exhibit AP as a result. These results present a partial mechanism for the effect of adolescent IA on AP, expand research on the influencing factors of AP, and provide new explanations for the complex psychological causes of AP and a reference for interventions for adolescent AP.

Despite the significance of the results of this study, there are some limitations. First, the sample size was limited and included only adolescents from a high school and university in Southwest China, and the results may not be generalizable to all adolescents. Future research should expand the scope of the research population. Second, this study used only self-reported measurement methods, and the participants may have had response bias. Future research should include evaluations from others for objective and accurate assessments.

In conclusion, based on the results of this study, aiming to effectively intervene academic procrastination, schools and families should provide more variable entertainment choices to avoid internet addiction. In order to ensure the progress of study, Teachers and parents also need to pay attention to students’ internet use time and emotional state, so as to avoid academic procrastination because of the decline of self-control and anxiety (e.g., negative emotion). Schools can improve individuals self-efficacy by conducting relevant courses, psychological activities and other means to enhance self-control (Chen et al., 2024), conduct regular interviews, investigate students’ life, and intervene students’ anxiety as soon as possible (Ju and Lv, 2022), so as to effectively deal with academic procrastination.

## Conclusion

This study found that Internet addiction not only has a direct effect on academic procrastination, but also self-control and anxiety can partially mediate the relationship between Internet addiction and academic procrastination. In addition, self-control and anxiety also play a serial mediating role in the relationship between internet addiction on academic procrastination.

## Data Availability

The original contributions presented in the study are included in the article/supplementary material, further inquiries can be directed to the corresponding authors.
